# Annual Meeting

**Published:** 1920-02-14

**Authors:** 


### THE NURSES' CO-OPERATION.

### Annual Meeting.

THE annual meeting of the Nurses' Co-operation was held at the Howard de Walden Nursing Home on Friday, February 6, when Dr. Low presided over a large and representative gathering, being supported by Sir Henry Burdett, Dr. Turney, and Dr. Giles.

**The Chairman,** in presenting the annual report and statement of accounts, said that since 1890, the year in which the Nurses' Co-operation was founded, over one million sterling had been earned by the nurses. Unfortunately, for some time past there had been very considerable friction between the members and some of the nurses' representatives on the Committee of Management. Some of the nurses' representatives had the idea that profit was being made out of the Co-operation, and he would like to point out that no member was allowed to make any profit whatever. Some difficulty had been felt with regard to nurses joining as members owing to the very strict rule in the Memorandum. The point had been raised so far back as 1891, when the Board of Trade refused to sanction a scheme suggested by Sir Capel Slaughter. Counsel's opinion had been taken, and he had advised that it would be better to leave the Memorandum and Articles of Association as they originally stood. When he told them that the Memorandum laid it down that not a single penny from the funds of the Co-operation should be paid out to any of the nurses, those present would realise that if nurses were admitted as members, such membership would be but an empty compliment. That, however, had been a bone of contention among the nurses' representatives; not only that, but they had gone to work and sent out circulars charging the Co-operation with double dealing. That charge had been answered in no uncertain manner, for as a result of the circular sent out to working nurses in order that they might have an opportunity of saying whether they supported the present constitution or not, out of a total of 493 nurses on the nursing staff, 400 had replied in the affirmative, and only eleven in the negative. Seventeen nurses had refused to uphold the present constitution, seventeen refused to sign, and two remained neutral, two wished for alterations. Ten letters had been received stating reasons for not signing, and one card was withdrawn. Fifty nurses had not replied at all, including twenty-four residing abroad, so that the percentage in favour of the present constitution was very great indeed. (Applause.) Members had also received a very welcome and complimentary letter, signed by 161 nurses, expressing their loyalty to the Co-operation. (Cheers.)

**Lady Makins** then presented the report of the scrutineers on the ballot taken from nurses, which showed that out of a total of 493 papers sent out, 390 had been returned, the four nurses' representatives elected being: Nurse Bremner (251 votes), Nurse Paterson (226), Nurse Mitchell (219), and Nurse Murray (211).

The elections having been approved,

**The Chairman** read many letters and telegrams regretting inability to be present at the meeting, Sister Ward expressing the hope that the meeting would press for the resignation of all agitators from the staff, a similar wish being also expressed in other letters and telegrams.

The LADY SUPERINTENDENT's report on fees received by nurses during the year, showed that over one hundred nurses had earned amounts varying from £50 to £140, one nurse having earned £222.

**Sir Henry Burdett** who was received with enthusiasm and cheered throughout the proceedings, then addressed the meeting. He said that, though he had had much to do with the origination of the Co-operation, yet he had not for many years taken an active part in its management or affairs. He was, however, present by request because of the unfortunate circumstances which had arisen and the differences of opinion which had occurred. Such differences should certainly not have occurred if those who were concerned had first of all made themselves acquainted with the constitution of the organisation under which the Co-operation had worked so happily, so successfully, and so profitably for nearly thirty years. It seemed hardly credible that they as business women and working nurses, with the Co-operation protecting their interests and making over one million pounds in under thirty years, should have those differences in the twenty-ninth year of its existence. It might be due to one of those many after-the-war effects from which the nation was at present suffering, and which had a great deal more to do with the exciting intrusions into

**The Nurses' Co-operation**—(*continued*).

ordinary business affairs than many people supposed. The Co-operation had been formed with the object of protecting working nurses, and of seeing that those who employed and sent out staff nurses should not interfere with the working nurses or undersell them. That object had been attained, and they, one and all, owed a great debt to all those members—retired nurses and others—who took a very great interest in the welfare of nurses and spent their time and energies to an extent which many did not realise, in loyal, devoted, continuous, unselfish service. That service was rendered with one desire only, namely, to enable the working nurse to be strong enough to defend herself and to take all her earning with the minimum deduction due only to the actual out-of-pocket expenses incidental to the maintenance of a great organisation such as theirs. As the Chairman had said, nobody could be a member who made a farthing of profit out of the earnings of nurses, and both they and the public would, therefore, understand that what had been said in regard to members was not only unwarranted, but quite unworthy of the people who had uttered those libels. (Hear, hear.) At the inception of the Co-operation a great difficulty had had to be overcome, and that brought about the existence of the member. Money had to be found in order to carry on, for none of its founders, being who they were, could give their names to a Co-operation unless provision was made that it should continue, and that, if the management were good, it should have a reasonable and fair chance, and nurses should be ensured all the benefits that would follow from that good management. The desire was that no nurse should be charged more than 7½ per cent. As a further instance of the difficulties and opposition which had to be overcome, Sir Henry quoted from one of the many letters he had received at the time of the formation of the Co-operation. The nurse in question, a very distinguished lady in the profession, had informed him bluntly that if he did not drop the Co-operation and take care that it did not proceed, he would be " put into Coventry " by every decent matron in the land. That, of course, was long ago, and had not come to pass. He congratulated, and was proud of, those who, having come into the possession of their own property, had had the wisdom, the courage, and the unanimity to stand shoulder to shoulder. That was the way to make the Co-operation prosper, to extend its activities and to bring some money to the nurses. They could be perfectly certain that so long as they continued in that direction they would thrive, because they deserved to. But they must bear in mind that increased income meant increased work for the clerical staff. Nurses very often did not realise the amount of work which devolved upon the very loyal staff of the Co-operation, but he was sure they, one and all, very much appreciated the work they knew was so well done for them. Now that they had overcome their difficulty and were earning £60,000 a year, surely they ought to go forward as a united body and support those who so loyally supported them. (Cheers.) He hoped they would so go forward and prosper. They would then have every justification for feeling that the best day's work they ever did was when they joined the Nurses' Co-operation, and that the worst day's work they could do, as working nurses, would be to leave it. (Applause.)

**Dr. Turney** (late Chairman of the Co-operation) said he felt somewhat in the position of that animal once described as being wicked enough to defend itself when attacked. In any case, he felt he owed an apology to the very considerable and representative body of nurses before him for having felt any doubt as to the true feeling of the general body of nurses. Wonderful testimony had been given as to the stability and qualities shown by the nursing profession, in that, in spite of a great deal which had been said which might have led to unsettlement, the nurses had remained practically, though not literally, unanimous in their adherence to the Co-operation. (Cheers.) Innuendoes had been multiplied and carried on for a long period of time, and, although one knew there was nothing behind them, yet such innuendoes had a certain effect. Therefore he could only say how glad he was to have that most remarkable testimony on the part of nurses, and to know that they had replied in no uncertain voice. With that answer the time had come when no more should be heard of those innuendoes. (Hear, hear.)

A question having been raised with regard to the source of the money subscribed to set the Co-operation going,

**Sir Henry Burdett** said that at the foundation of the Co-operation in 1891 financial aid had been given by Mr. Cheston to the extent of £100, by Mr. Slaughter to the extent of £200, and those gentlemen had subsequently given those sums of money to the Co-operation. Miss Belcher, the originator of the proposal for a Co-operation—for they owed it to the foresight of a nurse, not of a mere man—lent £75, Miss Ward lent £50, Miss Napper £24, and Miss R. Napper £50. The loans from those four nurses had been repaid on November 10, 1892, *plus* 4 per cent. interest. Further, Miss Morten, the first Honorary Secretary of the Co-operation, and one who had worked very hard for nurses throughout her life, spent an immense amount of time, aided by her father (a lawyer) in formulating a workable scheme, which had been taken up and carried through, as his hearers all knew, to a very successful issue.

A vote of thanks was then heartily accorded the Chairman, Dr. Turney, Sir Henry Burdett, and the Honorary Members.

The Chairman, in briefly returning thanks, hoped that their motto for the future would be " Peace." (Applause.)

A vote of thanks having been cordially passed to the members of the Home Committee also, together with a vote of confidence in Mrs. Crowe (the Secretary), the proceedings terminated.

